# Treatment of bipolar depression with minocycline and/or aspirin: an adaptive, 2×2 double-blind, randomized, placebo-controlled, phase IIA clinical trial

**DOI:** 10.1038/s41398-017-0073-7

**Published:** 2018-01-24

**Authors:** Jonathan B. Savitz, T. Kent Teague, Masaya Misaki, Matt Macaluso, Brent E. Wurfel, Matt Meyer, Douglas Drevets, William Yates, Ondria Gleason, Wayne C. Drevets, Sheldon H. Preskorn

**Affiliations:** 1 Laureate Institute for Brain Research, Tulsa, OK USA; 2 Faculty of Community Medicine, University of Tulsa, Tulsa, OK USA; 3 Department of Surgery, University of Oklahoma College of Medicine, Tulsa, OK USA; 4 Department of Psychiatry, University of Oklahoma College of Medicine, Tulsa, OK USA; 5 Department of Pharmaceutical Sciences, University of Oklahoma College of Pharmacy, Tulsa, OK USA; 6 Department of Biochemistry and Microbiology, Oklahoma State University Center for Health Sciences, Tulsa, OK USA; 7 Department of Psychiatry and Clinical Trials Unit, University of Kansas School of Medicine, Wichita, Kansas USA; 8 Department of Medicine, Oklahoma University Health Sciences Center, and Oklahoma City VAMC, Oklahoma City, Oklahoma USA; 9 Janssen Research and Development, LLC of Johnson and Johnson, Inc., Titusville, NJ USA

## Abstract

Given evidence of chronic inflammation in bipolar disorder (BD), we tested the efficacy of aspirin and minocycline as augmentation therapy for bipolar depression. Ninety-nine depressed outpatients with BD were enrolled in a 6 week, double-blind, placebo-controlled trial, and randomized to one of four groups: active minocycline (100 mg b.i.d.) + active aspirin (81 mg b.i.d.) (M + A); active minocycline + placebo aspirin (M + P); placebo-minocycline + active aspirin (A + P); and placebo-minocycline + placebo aspirin (P + P). A blinded interim analysis mid-way through the study led to the dropping of the M + P and A + P arms from further enrollment giving numbers per group who were included in the final analysis of: 30 (M + A), 18 (M + P), 19 (A + P), and 28 (P + P). When the study started, there were three primary outcome measures. Based on the results of the interim analysis, the primary outcome variable, response to treatment as defined by >50% decrease in Montgomery–Äsberg Depression Rating Scale (MADRS) score was maintained. The other two (i.e., the change in mean MADRS score from baseline to end of study and the remission rate, with remission being defined as a score of <11 on the MADRS) were reduced to exploratory outcome measures because the interim analysis indicated that the study was adequately powered to test differences in response rate but not the mean change in MADRS scores or remission rates. CRP and IL-6 were assayed to measure inflammation. Urinary thromboxane B2 (11-D-TXB_2_) concentrations, which were significantly increased at baseline in the combined BD sample (*n* = 90) vs. a healthy control group (*n* = 27), served as an indirect marker of cyclooxygenase (COX) activity. In a two-group analysis, the M + A group showed a greater response rate than the P + P group (*p*(one-tailed) = 0.034, OR = 2.93, NNT = 4.7). When all four arms were included in the analysis, there was a main effect of aspirin on treatment response that was driven by both the M + A and the A + P groups (*p*(two-tailed) = 0.019, OR = 3.67, NNT = 4.0). Additionally, there was a significant 3-way interaction between aspirin, minocycline, and IL-6, indicating that response to minocycline was significantly greater in participants in the M + P group with higher IL-6 concentrations. Further, participants in the M + P group who responded to treatment had significantly greater decreases in IL-6 levels between baseline and visit 7 vs. non-responders. Regarding the exploratory outcomes, there was a main effect for aspirin on the remission rate (*χ*_1_^2^ = 4.14, *p*(2t) = 0.04, OR = 2.52, NNT = 8.0). There was no significant main effect of aspirin or minocycline on the mean change in MADRS score across visits. Aspirin and minocycline may be efficacious adjunctive treatments for bipolar depression. Given their potential import, additional studies to confirm and extend these findings are warranted.

## Introduction

The treatment of bipolar depression is a major clinical challenge and no conventional antidepressants have been approved by the FDA for the short-term treatment of bipolar depression^1^. Only three pharmacotherapies are FDA-approved for bipolar depression; the combination of olanzapine and fluoxetine (OF), quetiapine monotherapy (QTP), and lurasidone as a monotherapy or adjunctive therapy. These treatments produce numbers needed to treat (NNT) for response of 4 for OF, 6 for QTP, and 5–7 for lurasidone, respectively^2^, and all three produce significant side-effects with numbers need to harm (NNH) of 6 for OF (e.g., weight gain), 5 for QTP (e.g., sedation), and 15–16 for lurasidone (e.g., akathisia and nausea), respectively^2^. Commonly used agents which do not have a labeled indication for treatment of the depressed phase of bipolar disorder, such as lamotrigine (NNT = 12; NNH = 37) and lithium (NNT = 15; NNH = 38), are modestly efficacious^2, 3^. Thus, new classes of medication are needed.

Given that an inflammatory-like state exists in a subgroup of patients with BD, there is increasing interest in the therapeutic potential of immune-modulating medications^4–7^. Two particularly promising candidates are minocycline and low-dose aspirin, as reviewed in the initial published protocol^8^ and summarized here: both medications are well-tolerated, even with long-term use, well absorbed and brain penetrant, and likely exert anti-inflammatory effects in the brain and the periphery.

Aspirin inhibits cyclooxygenase-1 (COX-1) and acetylates COX-2, blocking the conversion of arachidonic acid to prostaglandins and thromboxane A2. At the low dose used in this study, aspirin preferentially inhibits COX-1 while at higher doses it additionally reduces COX-2 function. COX-1 is predominantly expressed by microglia and macrophages while COX-2 is predominantly localized to neurons^9^. Importantly, preclinical evidence suggests that inhibition of COX-1 is neuroprotective after intracerebroventricular administration of lipopolysaccharide (LPS) whereas inhibition of COX-2 is detrimental, increasing leukocyte recruitment into the brain and exacerbating tissue damage^10^. Notably, a pharmacoepidemiological study demonstrated that individuals treated with lithium and low dose aspirin (≤80 mg/day) were less likely to have a medication event (medication switch or dose change) whereas high-dose aspirin, non-selective NSAIDs, and glucocorticoids were associated with an increase in medication events^11^. While there are no published controlled clinical trials of aspirin as an antidepressant, an open-label study reported aspirin increased the speed of response to SSRIs^12^, and an epidemiological study reported that aspirin protected against depression in older men with elevated levels of homocysteine^13^.

Minocycline, a tetracycline antibiotic, exerts a variety of biological actions that are independent of its anti-microbial activity, such as anti-inflammatory and anti-apoptotic activities, and inhibition of proteolysis, angiogenesis, and tumor metastasis. Minocycline modulates immune function via multiple mechanisms—for instance, inhibiting the activation, migration, and/or proliferation of T-cells, neutrophils, and microglia, inhibiting the release of pro-inflammatory cytokines, and increasing the release of anti-inflammatory and anti-apoptotic molecules^14^. In preclinical models, minocycline exerts neuroprotective effects, including reversing inflammation-induced inhibition of neural stem cell proliferation^15^ and reducing lesion size and/or demyelination in neurological disorders, such as Huntington’s disease^16^. Antidepressant-like effects have been reported in rodents^17^ and anecdotally in humans^18, 19^, as well as in an open-label trial for bipolar depression ^20^. In addition, a double-blind, placebo-controlled, randomized trial of minocycline for mild-to-moderate depression in HIV patients showed therapeutic effects^21^. No randomized controlled trial has been published in patients with primary mood disorders.

Here we perform the first randomized, placebo-controlled trial of aspirin and minocycline to assess their efficacy as adjunctive treatments for bipolar depression alone and together. Given the well-established safety profiles of these drugs, a 2 × 2 design was chosen for study as it permitted an opportunity to test two mechanistically different anti-inflammatory agents. Second, it allowed an opportunity to test for whether these mechanisms augmented or interfered with each other. If either aspirin or minocycline proved to be ineffective while the other agent was effective and both were well tolerated, then the two cells with the effective agent (i.e., effective agent plus placebo and effective agent plus the ineffective agent) could be combined and compared to the double placebo group combined with group which received the ineffective drug plus placebo, essentially doubling the statistical power of the study. On the other hand, if the two drugs were found to either augment or interfere with each other, then combining the groups to increase power would not be possible but valuable clinical information would be obtained.

## Methods

### Study design

**Fig. 1:**
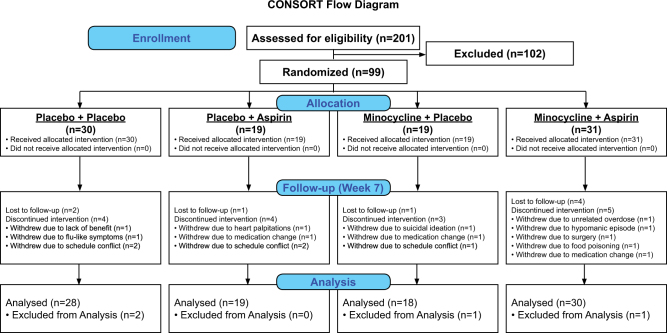
CONSORT flow diagram showing the number of individuals assessed for eligibility in person, the number of participants randomized to each group, the number of individuals lost to follow-up at visit 7 (week 6) and the number of individuals included in the statistical analyses

This was a multi-site, double-blind, placebo-controlled clinical trial in which as adjunctive therapy to existing treatment, participants initially were randomized (1:1:1:1) to one of 4 groups: (a) active minocycline (100 mg b.i.d., p.o.) + active aspirin (81 mg b.i.d., p.o.) (M + A); (b) active minocycline (100 mg b.i.d., p.o.) + placebo aspirin (M + P); (c) placebo minocycline + active aspirin (81 mg b.i.d., p.o.) (A + P), and (d) placebo minocycline + placebo aspirin (P + P). At the mid-way point when 60 patients had completed the study, an adaptive design approach was taken to perform a blind interim analysis to test for futility. This blind interim analysis, performed by a statistician, found that two groups appeared to be separating from each other. A new power calculation was performed using the data from these two groups (i.e., the potential effect size and variability) and determined that 30 participants in these two cells would yield sufficient power to test for a difference in their respective response rates. In contrast, sufficient power was not available to detect group differences in the mean change in MADRS scores over time. Subsequent to the interim analysis, WCD, who did not assess participants and was not at any of the sites at this point in the study, was unblinded and observed that the M + A group was separating from the P + P group. This information was not conveyed to the clinical teams conducting the study. In addition, at this point in the study, it was apparent that we could only enroll 100 participants rather than the original goal of 120 and stay within our study budget. For these reasons, the design was adapted by stopping future enrollment into the A + P and M + P groups and instead randomization of all future participants (projected to be 36 based on the historical rate of participant accrual in the study) was to the M + A or P + P groups, resulting in the numbers per group included in the final statistical analyses of: 30 (M + A), 18 (M + P), 19 (A + P), and 28 (P + P). See CONSORT diagram (Fig. 1). The reason the projected number of additional subjects was 36 rather than 40 is because four more subjects (i.e., a total of 64) were already enrolled in the study but had not completed the study when the interim analysis was performed.

Parenthetically, adaptive trial designs allow for the modification of aspects of a study in a real-time, data-driven manner, and thus can greatly enhance the efficacy of early drug development^22^. The adaptive design is usually employed in early studies to test either many doses of a drug or different combinations or schedules of administration (e.g., oncology), and then is followed by at least one confirmatory conventional study for a new drug application to a regulatory agency, such as the Food and Drug Administration.

The duration of the trial was 6 weeks and comprised 7 visits (baseline and weekly follow-up, Figure S1). The study was conducted at three sites: the Laureate Institute for Brain Research (LIBR) in Tulsa, the University of Oklahoma College of Medicine (OU), Tulsa, and the University of Kansas School of Medicine (KUSM-W) in Wichita. The Western IRB was used as the central IRB for the three sites and this use was approved by the IRBs of OU and KUSM-W. The study protocol was published prior to the trial^8^.

### Participants

Participants were recruited from psychiatric clinics associated with the study sites, as well as the general community through radio and print advertisement. The treatment that the participants received prior to study enrollment was determined by their treating clinician.

#### Inclusion criteria

(a) meeting DSM-IV-TR criteria for BD I (*n* = 37), BD II (*n* = 57), or BD NOS (*n* = 5) based on the MINI-Plus and a clinical interview by a study psychiatrist; (b) current major depressive episode of ≥4 weeks duration, (c) at least moderately depressed, i.e., a Quick Inventory of Depressive Symptomatology (QID-C16) score ≥10; (d) stable regimen of medication for ≥4 weeks prior to enrollment (if receiving therapy), (e) 18–65 years of age. Exclusion criteria are detailed in the supplement and a summary of the psychiatric medications taken by the participants during the trial appears in Table S1.

The study was approved by the western IRB and participants provided written informed consent. The trial was publically registered (www.clinicaltrials.gov): NCT01429272. Minor amendments to the published pre-study protocol are as follows: (a) The threshold for depression severity originally was a score of at least 18 on the HAM-D 17. We switched from the HAM-D to the QID-C 16 because of its better parametric characteristics but the severity threshold remained identical: a score of 10 on the QID-C 16 is equivalent to an 18 on the HAM-D 17. (b) The proposed age range of 18–55 was altered to 18–65 to facilitate recruitment. (c) Similarly, in order to facilitate recruitment, we broadened the entrance criteria to allow for the inclusion of participants with a diagnosis of BD NOS. (d) A blind interim analysis was performed leading to the dropping of two arms from the study. (e) We used more rigorous criteria for response and remission than was specified in the protocol. The MADRS-based criteria for response (>50% decrease in MADRS scores) and remission (MADRS score of <11) were retained as these are standardly used in the field^23^. However, in the protocol we stated that these criteria would apply at the final visit of the participant whereas here we required the 50% decrease in MADRS and/or the MADRS score of <11 to be present for the final two consecutive visits in line with recent FDA advice. (f) Time of blood draw was not used as a covariate in the immune analyses because this information was not available for all participants.

To assess and enhance participant adherence to study medication, participants were given an information sheet to take home detailing the procedure to be followed in the case of a missed dose, and requesting that this information be recorded for the investigators. Returned trial medication was audited. Participants were permitted to miss no >50% of their study medications in a single week on more than one occasion. Non-adherent participants received additional counseling about the need for adherence beyond what was routinely given each week. A second episode of non-adherence would have resulted in withdrawal from the study but did not occur. We also obtained a post-study measure of medication adherence for the participants receiving aspirin, i.e., urinary concentrations of thromboxane B2 (11-D-TXB_2_), a downstream metabolite of prostaglandin H2, that is robustly decreased by treatment with aspirin^24^.

### Randomization and masking

Participants were randomly assigned to one of the four arms in a double-blind fashion via a central database hosted at LIBR. Randomization was conducted according to CONSORT guidelines using block permutations. Randomization codes were generated by a computer-based random number generator and were held by LIBR staff not involved in the trial until study completion. The investigators and participants were blind to the treatment allocation. Blindness was maintained by ensuring that the packaging, appearance and color of the minocycline, aspirin, and placebo capsules were identical. Commercial minocycline and aspirin tablets were purchased by the manufacturer (Wedgewood Pharmacy, Swedesboro, NJ) and over-encapsulated. Matching placebo capsules containing lactose were produced.

### Biological samples

Serum samples were obtained at baseline (*n* = 90) and visit 7 (*n* = 80) to measure inflammatory biomarkers. CRP and interleukin-6 (IL-6), were measured using a Meso Scale Discovery (MSD) QuickPlex SQ 120 instrument and MSD V-PLEX assays (CRP: LLOQ = 0.05 mg/L; CV = 1.9%; IL-6: LLOQ = 0.04 pg/mL; CV = 3.6%). A spot morning urine was taken and a 11-Dehydro Thromboxane B2 EIA kit (Cayman Chemical Company; Ann Arbor, MI) used to quantify 11-D-TXB_2_ (LLOQ = 46 pg/mL; CV = 4.3%), an indirect marker of COX activity that has been used to measure adherence to aspirin therapy in cardiovascular and cerebrovascular diseases^24^. For purposes of comparison, we also obtained two 11-D-TXB_2_ measures from a healthy control sample (*n* = 27), 6 weeks apart.

### Outcome measures

Based on the results of the interim analysis (see Study Design above), two of three original primary outcome measures, i.e., the change in MADRS scores across visits and remission rate were reduced to exploratory outcome measures. The third original primary outcome variable, i.e., response to treatment was maintained. Sustained response to treatment was defined as a >50% reduction^23^ in Montgomery–Äsberg Depression Rating Scale (MADRS) for the final two consecutive visits of each participant^25^. The exploratory outcome measure, remission, was defined as a post-treatment MADRS score of <11^23^ for the final two consecutive visits^25^. Other exploratory outcome measures were the mean change in MADRS score, Clinical Global Impression Improvement (CGI-I) score, and Hamilton Anxiety Scale (HAM-A) score over time.

Adverse events were recorded at each visit and were posed as open-ended questions about any issues with the trial or the study medication in accordance with standard FDA guidance for the execution of such clinical trials (Table S2). Safety was monitored by the study PI, a biweekly consensus meeting of the principal investigator (PI) and study staff at each site, and a Data, Safety and Monitoring Board (DSMB) which met biannually.

### Hypotheses

#### Primary hypothesis

##### *Hypothesis 1:*

Participants in the M + A group will show a greater sustained response rate than participants in the P + P group.

#### Secondary hypotheses

##### *Hypothesis 2:*

Across all four groups, participants receiving aspirin and/or minocycline will show a greater response rate than participants in the P + P group.

##### *Hypothesis 3:*

The efficacy of aspirin and/or minocycline treatment will be predicted by baseline inflammation such that patients receiving active treatment with higher levels of IL-6 and CRP will show a greater response rate to treatment. Additionally, participants who show a greater decrease in IL-6 and CRP concentrations between visits 1 and 7 will show a greater response to treatment.

### Exploratory analyses (EA)

(EA 1). Compared to the P + P group, patients receiving aspirin and/or minocycline will show a greater remission rate.

(EA 2). Compared to the P + P group, patients receiving aspirin and/or minocycline will show a greater decrease in mean MADRS scores over time.

(EA 3). Compared to the P + P group, patients receiving aspirin and/or minocycline will show a greater decrease in CGI-I scores over time.

(EA 4). Compared to the P+P group, patients receiving aspirin and/or minocycline will show a greater decrease in mean HAM-A scores over time.

### Statistical analysis

Analyses were performed with R^26^. Analysis of Variance (ANOVA) or the *χ*^2^-test was performed to test for baseline differences in demographic and clinical variables across the four treatment arms. Concurrent medications were coded into four classes, i.e., antidepressants, anticonvulsants, antipsychotics, and anxiolytics (Table S1). There was no significant difference between groups in numbers of participants per medication class, age, sex, and body mass index (BMI). Results also did not differ significantly across sites.

Between group differences in response rate were tested with logistic regression with the analysis of deviance test. To evaluate the effects of baseline inflammation on response rate, IL-6 and CRP were entered as additional binary (i.e., low vs. high based on a median split) variables. Variables were selected as regressors according to whether their inclusion improved the model measured by the Akaike information criterion (AIC) score. Based on the AIC score, no covariates (i.e., age, sex, body mass index (BMI), medication class nor study site) were included in the models as covariate regressors for the logistic regression analyses.

For the post hoc analyses, differences in remission rate were tested with logistic regression with the analysis of deviance test. Linear Mixed Effect (LME) model analyses were performed to measure the temporal change in mood ratings (MADRS, CGI-I, and HAM-A scores) across visits. Age, sex, and BMI were included as fixed effects based on the AIC. Random effects included subject and autoregressive covariance structure across visits within subject (see Supplement).

For the primary and secondary hypotheses, we used a standard statistical significance threshold of *p* < 0.05. For the exploratory analyses, we used a Bonferroni-corrected statistical threshold of *p* < 0.013 (to correct for four tests).

### Role of the funding source

The funders of the study had no role in the study design, data collection, data analysis, data interpretation, or writing of the report. The corresponding authors had full access to all the data in the study and had final responsibility for the decision to submit for publication.

## Results

Ninety-nine participants were randomized out of a total of 201 individuals who underwent in-person screening (Fig. 1). The recruitment period was from March 2012 to August 2015. Initially, we believed 100 participants had been randomized and hence closed the study; however, one person was erroneously counted twice. Seventy-five participants completed all visits. Four participants did not complete any post-baseline visits and were excluded from the analyses. The difference in urine 11-D-TXB_2_ concentrations between baseline and visit 7 demonstrates target engagement of COX by aspirin in the groups that received treatment with aspirin (Figure S2). Baseline characteristics of the sample and the number of participants who completed each visit are shown in Tables 1, 2, respectively.

**Table 1:** Demographic and clinical characteristics of the M + A, M + P, A + P, and P + P groups at baseline

	M + A	M + P	A + P	P + P	Statistic
*N* (BD)	31	19	19	30	—
*N* (I/II/NOS)	11/20/0	7/10/2	5/13/1	14/14/2	*χ*^2^(6) = 5.54, *p* = 0.476
Age	40.8 ± 9.7	44.8 ± 8.7	40.6 ± 10.2	40.8 ± 10.4	*F*(3,95) = 0.88, *p* = 0.455
Sex (% *F*)	84	68	68	73	*χ*^2^(3) = 2.20, *p* = 0.531
BMI	31.8 ± 8.6	31.1 ± 5.0	32.1 ± 7.2	30.9 ± 10.1	*F*(3,95) = 0.11, *p* = 0.952
MADRS	28.0 ± 5.7	27.2 ± 5.2	25.9 ± 6.5	29.2 ± 6.2	*F*(3,95) = 1.23, *p* = 0.302
HAM-A	19.9 ± 7.7	19.6 ± 7.3	19.0 ± 7.2	19.9 ± 7.3	*F*(3,94) = 0.08, *p* = 0.972
YMRS	4.7 ± 2.2	3.3 ± 2.4	5.6 ± 3.4	3.9 ± 2.5	*F*(3,95) = 3.13, *p* = 0.029*
CGI severity	4.1 ± 0.5	4.3 ± 0.5	4.2 ± 0.5	4.3 ± 0.5	*F*(3,94) = 0.93, *p* = 0.428
CRP (mg/L)	8.2 ± 12.9	4.3 ± 5.5	4.5 ± 4.9	4.4 ± 5.3	*F*(3,86) = 1.30, *p* = 0.281
IL-6 (pg/mL)	1.0 ± 0.7	0.9 ± 0.5	1.1 ± 0.7	0.9 ± 0.5	*F*(3,86) = 0.44, *p* = 0.724
TXB_2_ (pg/mL)	2665 ± 2834	2730 ± 2449	4765 ± 3704	5038 ± 4626	*F*(3,86) = 2.96, *p* = 0.037*

*BMI* body mass index, *MADRS* Montgomery–Äsberg Depression Rating Scale, *HAM-A* hamilton anxiety scale, *YMRS* young mania rating scale, *CGI* clinical global impressions scale, *CRP* c-reactive protein, *IL-6* interleukin-6, *11-D-TXB*_*2*_ Thromboxane B2

*p < 0.05

**Table 2:** MADRS scores and inflammatory biomarker concentrations at visits 1–7 (Mean ± SD)

Outcome/visit	Group	*N*	MADRS	*T*; *p*-value (vs. P + P)	CRP (mg/L)	IL-6 (pg/mL)	11-D-TXB_2_ (pg/mL)
V1	M + A	31	28.0 ± 5.7	1.03; 0.308	8.2 ± 12.9	1.0 ± 0.7	2665 ± 2834
	M + P	19	27.2 ± 5.2	0.87; 0.386	4.3 ± 5.5	0.9 ± 0.5	2730 ± 2449
	A + P	19	25.9 ± 6.5	1.84; 0.069	4.5 ± 4.9	1.1 ± 0.7	4765 ± 3704
	P + P	30	29.2 ± 6.2		4.4 ± 5.3	0.9 ± 0.5	5038 ± 4626
V2	M + A	30	21.5 ± 7.1	1.92; 0.058	—	—	—
	M + P	18	21.4 ± 8.3	1.34; 0.185	—	—	—
	A + P	19	21.6 ± 7.2	1.44; 0.154	—	—	—
	P + P	27	25.0 ± 7.4		—	—	—
V3	M + A	27	20.1 ± 6.8	1.34; 0.183	—	—	—
	M + P	17	19.8 ± 9.5	1.07; 0.288	—	—	—
	A + P	19	17.6 ± 7.3	2.08; 0.040*	—	—	—
	P + P	27	23.0 ± 9.7		—	—	—
V4	M + A	27	17.8 ± 8.1	1.63; 0.108	—	—	—
	M + P	16	16.9 ± 8.5	0.54; 0.589	—	—	—
	A + P	18	15.9 ± 6.0	1.56; 0.122	—	—	—
	P + P	27	20.3 ± 9.2		—	—	—
V5	M + A	27	15.9 ± 9.1	1.02; 0.311	—	—	—
	M + P	16	17.7 ± 9.0	0.11; 0.912	—	—	—
	A + P	16	15.2 ± 9.3	1.01; 0.318	—	—	—
	P + P	26	18.4 ± 8.5		—	—	—
V6	M + A	24	15.5 ± 9.1	0.75; 0.455	—	—	—
	M + P	16	16.7 ± 7.6	0.12; 0.908	—	—	—
	A + P	14	13.7 ± 9.3	1.16; 0.251	—	—	—
	P + P	25	17.6 ± 9.3		—	—	—
V7	M + A	22	14.5 ± 8.9	0.50; 0.619	10.3 ± 20.4	1.0 ± 0.5	1867 ± 2238
	M + P	15	15.5 ± 8.0	0.09; 0.928	2.2 ± 2.2	0.8 ± 0.3	3323 ± 3107
	A + P	14	12.3 ± 8.4	1.12; 0.267	3.9 ± 4.4	0.9 ± 0.5	963 ± 724
	P + P	24	16.0 ± 9.6		5.0 ± 5.2	1.0 ± 0.7	4565 ± 3498

*MADRS* Montgomery–Äsberg Depression Rating Scale, *CRP* c-reactive protein, *IL-6* interleukin-6, *11-D-TXB*_*2*_ Thromboxane B_2_

*P < 0.05, uncorrected: ANOVA analysis controlling for sex, age, and BMI

One participant in the M + A group developed a hypomanic episode during the study and was withdrawn. Nevertheless, there was no difference between groups in the Young Mania Rating Scale (YMRS) scores at visit 7 (*F*_3,71_ = 1.44, *p* = 0.237). Parenthetically, the YMRS was used to prospectively test for any evidence that minocycline and/or aspirin could precipitate cycling into hypomania or mania. The dropout rate did not differ significantly across groups (*χ*^2^_3_ = 0.83, *p* = 0.842). There was no significant difference across the four arms in the number of participants who were being treated with antipsychotics (*χ*^2^_3_ = 0.41, *p* = 0.932), mood stabilizers (*χ*^2^_3_ = 0.45, *p* = 0.928), antidepressants (*χ*^2^_3_ = 0.87, *p* = 0.832), or anxiolytics (*χ*^2^_3_ = 2.01, *p* = 0.530) at study entry. Further, there was no difference between groups in the number of individuals with a diagnosis of BD I, BD II, and BD NOS (*χ*^2^_6_ = 5.54, *p* = 0.476). The adverse events per group appear in Table S2. The main results of the study are summarized in Table S3.

### **Hypothesis 1**

Participants receiving minocycline plus aspirin had a better response rate compared with participants receiving double placebo (*χ*_1_^2^ = 3.35, *p*(1t) = 0.034, odds ratio (OR) = 2.93, 95% confidence interval (CI) = 0.93–10.08, NNT = 4.7). When pre- vs. post interim analysis status was used as a covariate in the logistic regression, the M + A group still showed a significantly better response rate than the P + P over the entire trial (*χ*_1_^2^ = 3.35, *p*(1t) = 0.034). There was no main effect of pre- vs. post interim analysis status (*χ*_1_^2^ = 0.002, *p* = 0.961) or interaction between pre- vs. post interim analysis status and study arm (*χ*_1_^2^ = 1.35, *p* = 0.246). Based on these two analyses, no bias in the results was introduced by the interim analysis.

### **Hypothesis 2**

**Fig. 2:**
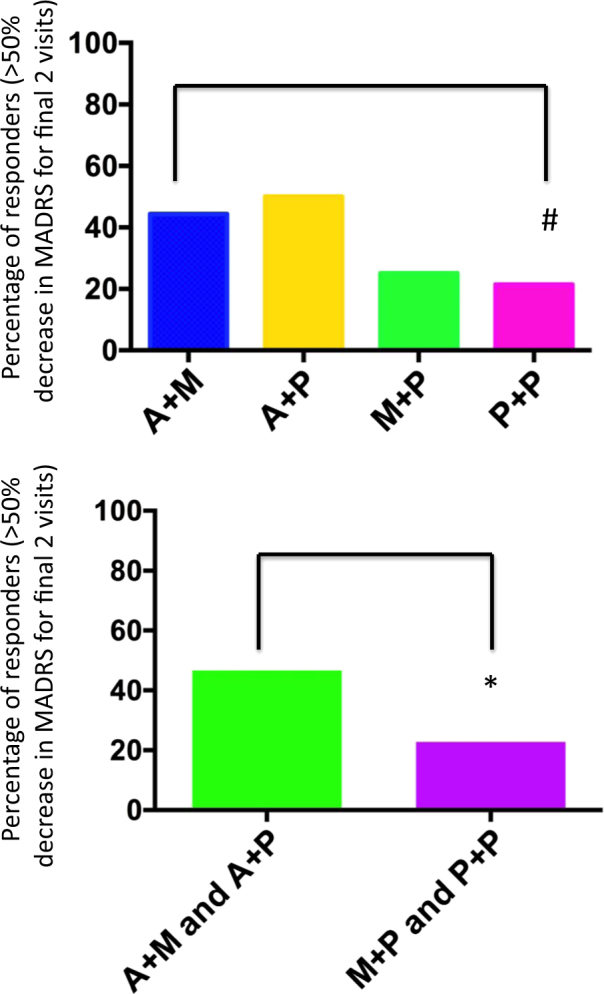
Percentage of responders (*y*-axis) in each of the 4 treatment groups shown individually (top panel) and the two aspirin groups (M + A and A + P) vs. the two non-aspirin groups (M + P and P + P) (bottom panel) Top panel: participants receiving minocycline plus aspirin showed a better response rate compared with participants receiving double placebo (*χ*_1_^2^ = 3.35, *p*(1t) = 0.034, odds ratio (OR) = 2.93, NNT = 4.7). Bottom panel: there was a significant main effect of aspirin on the clinical response rate (*χ*_1_^2^ = 5.52, *p*(2t) = 0.019, OR = 3.67) but no significant effect of minocycline (*χ*_1_^2^ = 0.01, *p* = 0.911) or interaction between aspirin and minocycline (*χ*_1_^2^ = 0.19, *p* = 0.659). The NNT to obtain a response to aspirin (M + A and A + P vs. M + P and P + P) was 4.2. The NNT for the M + A and A + P vs. the P + P comparison was 4.0. #*p* < 0.05 (one-tailed test); *p < 0.05 (two-tailed test)

There was a significant main effect of aspirin on the clinical response rate (*χ*_1_^2^ = 5.52, *p*(2t) = 0.019 with analysis of deviance test, (OR = 3.67, 95% CI = 1.03–14.06) but no significant effect of minocycline (*χ*_1_^2^ = 0.01, *p* = 0.911) or interaction between aspirin and minocycline (*χ*_1_^2^ = 0.19, *p* = 0.659) (Fig. 2). The NNT to obtain a response to aspirin (M + A and A + P vs. M + P and P + P) was 4.2. The NNT for the M + A and A + P vs. the P + P comparison was 4.0.

### **Hypothesis 3**

**Fig. 3:**
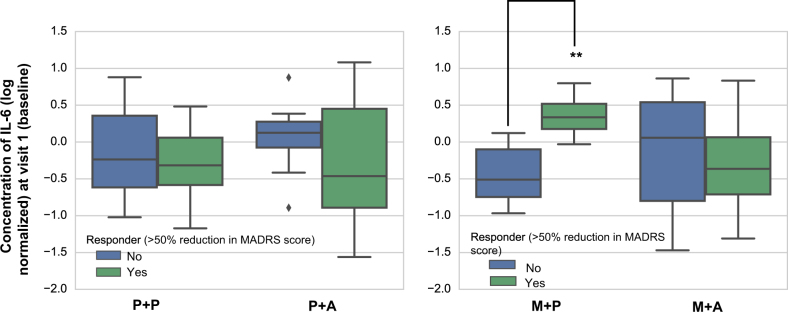
Box-and-whisker plots showing the 4 treatment groups divided into responders (green) and non-responders (blue) The box shows inter quartile range (IQR, i.e., 25–75%) around the median (line in a box) and the whisker shows data range (minimum to maximum values) except for outliers (diamonds). The natural logarithm of the serum IL-6 protein concentration is shown on the *y*-axis. There was a significant 3-way interaction between aspirin, minocycline, and IL-6 (*χ*^2^_1_ = 7.08, *p*(2t) = 0.008) on response rates. Follow-up analysis showed that participants in the M + P group with higher baseline IL-6 levels were more likely to be classified as treatment responders than participants in the M + P group with lower IL-6 levels (*χ*^2^_1_ = 7.72, *p*(2t) = 0.005). **p < 0.01 (two-tailed test); ◊ = outlier, defined as a data point outside of 75% + 1.5*IQR or 25%–1.5*IQR

There was a significant 3-way interaction between aspirin, minocycline, and IL-6 (*χ*^2^_1_ = 7.08, *p*(2t) = 0.008) on response rates. Follow-up analysis showed that participants in the M + P group with higher baseline IL-6 levels responded better to minocycline than participants in the M + P group with lower IL-6 concentrations (*χ*^2^_1_ = 7.72, *p*(2t) = 0.005, Fig. 3). Further, there was a significant interaction between the change in IL-6 concentrations and treatment response (*χ*^2^_3_ = 9.69, *p*(2t) = 0.001). Follow-up testing indicated that participants in the M + P group who responded to treatment had a significantly greater decrease in IL-6 levels between baseline and visit 7 compared with non-responders in the M + P group. There were no significant interactions between aspirin and CRP or minocycline and CRP, or the 3-way interaction between aspirin, minocycline, and CRP (all *p*’s > 0.1).

### Post hoc analyses

There was a main effect for aspirin on the remission rate that did not remain significant after Bonferroni correction (*χ*_1_^2^ = 4.14, *q*(2t) > 0.5, *p*(2t) = 0.042, uncorrected, OR = 2.52, CI = 0.56–12.29, Figure S3). There was no main effect for minocycline (*χ*_1_^2^ = 0.45, *p* = 0.503, uncorrected) or the interaction between aspirin and minocycline (*χ*_1_^2^ = 0.35, *p* = 0.554, uncorrected). The NNT for aspirin to obtain remission (M + A and A + P vs. M + P and P + P) was 6.5. The NNT for the M + A and A + P vs. P + P comparison was 8.0. There were no significant main effects of aspirin or minocycline on the mean change in MADRS, CGI-I, or HAM-A scores from baseline, nor were there significant interactions between aspirin, minocycline, and visit (Figures S4–S6).

## Discussion

This is the first randomized controlled trial of adjunctive minocycline and/or aspirin for the treatment of bipolar depression. There were two principal findings.

First, consistent with our findings from the interim analysis, there was a significantly greater sustained clinical response rate in the M + A group (44%) vs. the P + P group (21%, NNT = 4.7) with participants receiving minocycline plus aspirin approximately twice as likely to show a sustained response vs. those individuals receiving double placebo. For this specific analysis, we performed a one-tailed test because of our a priori hypothesis (based on the interim analysis) that the M + A group would show a greater response rate than the P + P group. However, irrespective of whether a one or two-tailed test is performed, the large effect size (OR = 2.93) raises the possibility that the combination of minocycline and aspirin may be an efficacious adjunctive treatment for bipolar depression. Further studies are needed to test whether the combination of minocycline and aspirin exerts a synergistic effect that is superior to either drug alone.

Given the initial 2 × 2 design, by combining groups as proposed in the original published protocol, we also could test for the main effects of the aspirin alone (i.e., A + P) or together with minocycline (i.e., M + A) vs. P with or without minocycline (i.e., P + P and/or M + P). Similarly, we also could test for the main effects of the minocycline alone (i.e., M + P) or together with aspirin (i.e., M + A) vs. P with or without aspirin (i.e., P + P and/or A + P). When all four treatment arms were included in the analysis (hypothesis 2), there was a significant main effect of aspirin (A + P and M + A combined) on sustained response rates with NNTs of between 4 (for A + P and M + A vs. P + P) and 4.2 (for A + P and M + A vs. P + P and M + P), i.e., equal to or superior to currently approved treatments for bipolar depression.

The second main finding was that participants with higher baseline levels of IL-6 responded better to minocycline administration than patients with lower levels of inflammation (hypothesis 3). These data resemble findings from a recent clinical trial of the TNF inhibitor, infliximab, for treatment resistant depression in which no overall difference in the change in depression ratings was detected between treatment groups across time^27^. However, infliximab-treated patients with a baseline CRP concentration >5 mg/L had a greater decrease in depression ratings compared to the placebo group^27^. The results from both the infliximab and the current study suggest that anti-inflammatory agents may not be helpful for treating depressed patients who do not manifest inflammation. In addition, the minocycline-treated participants who showed a greater decrease in IL-6 levels between baseline and visit 7, also showed a larger reduction in depressive symptoms over the trial. This result is consistent with recent studies reporting that depressed patients who respond/remit to treatment with an SSRI or electroconvulsive therapy show a greater decrease in IL-6 levels over time compared with non-responders or remitters^28, 29^. Conceivably, IL-6 could be used in future to predict which patients are likely to respond to minocycline.

The absence of a significant interaction between the efficacy of aspirin treatment and baseline levels of CRP and/or IL-6 may reflect Type II error given the relatively small samples, but also may reflect the clinically non-significant anti-inflammatory effect of low-dose aspirin in autoimmune or other inflammatory disorders. Our results therefore suggest that the therapeutic effect of aspirin may be attributable to a still unknown mechanism, conceivably the effect of COX-1 inhibition on the arachidonic acid cascade^30^ or neurotrophic processes^31^. Arachidonic acid not only is released by unregulated phospholipase A2 activation associated with neuroinflammation, leading to excess production of COX-mediated inflammatory metabolites, but also is a second messenger released during neurotransmission via dopaminergic (D_2_), glutamatergic (NMDA), serotonergic (5-HT_2A_, 5-HT_2C_), and muscarinic (M_1_, M_3_, and M_5_) receptors, raising the possibility that inhibition of COX by aspirin may modulate disturbed neurotransmission in bipolar depression^30^. In this regard, this first report of higher baseline 11-D-TXB_2_ levels in the BD sample relative to the control sample (Figure S2) is noteworthy as it suggests that the activity of the arachidonic acid pathway is elevated in a subset of individuals with BD, consistent with previous hypotheses^30^.

Strengths of the study include: (a) the placebo-controlled assessment of two novel therapeutic agents, with the 2 × 2 design allowing us to evaluate potential additive and antagonistic drug interactions, (b) the use of peripheral blood biomarkers to assess the effect of inflammation on treatment outcome, (c) target engagement of aspirin (based on the pharmacodynamic effects of COX inhibition) in the aspirin groups, and (d) the representative nature of our sample for individuals with bipolar depression, many of whom were inadequately responsive to existing treatments, which remains a serious unmet medical need.

Although we attempted to assess the effects of adjunctive medications by broadly grouping medications by class, we did not control for differences in dose, pharmacokinetics, and drug–drug interactions. This is a limitation of the study.

In sum, the study provides preliminary support to the possibility that aspirin and minocycline can be efficacious adjunctive therapies for the treatment of bipolar depression. These findings should encourage further studies in larger samples perhaps using markers of inflammation as inclusion criteria to increase statistical power. Independent confirmation of the therapeutic efficacy of low-dose aspirin has significant potential to advance the treatment of depression given its global availability and affordability, and its relatively benign side-effect profile.

## Electronic supplementary material

Supplementary Data

Supplementary FigureS1

Supplementary FigureS2

Supplementary FigureS3

Supplementary FigureS4

Supplementary FigureS5

Supplementary FigureS6

Supplementary Figure Legends
