# Supplementary material for: Treatment of bipolar depression with minocycline and/or aspirin: an adaptive, 2×2 double-blind, randomized, placebo-controlled, phase IIA clinical trial
Source: Transl Psychiatry. 2018 Jan 24;8:27. doi: 10.1038/s41398-017-0073-7 (PMC5802452; doi:10.1038/s41398-017-0073-7)
Supplement: Supplementary file 1 — Supplementary Data [file 41398_2017_73_MOESM1_ESM.docx]

**Supplementary Data**

**Inclusion criteria**

(a) Males or females aged 18-65 who meet DSM-IV-TR criteria for BD (type I or II or NOS); (b) current depressive episode of ≥4 weeks duration; (c) minimum threshold of depression severity of 10 on the Quick Inventory of Depressive Symptomatology (QID-C16); (d) stable regimen of medication for ≥4 weeks prior to enrollment (if receiving therapy). Participants were allowed to may remain in psychotherapy or have no psychosocial intervention.

**Exclusion criteria**

(a) Illness onset after 40 years of age; (b) serious risk of suicide; (c) current delusions or hallucinations sufficient to interfere with the capacity to provide informed consent; (d) current manic symptoms of sufficient severity to pose a substantial risk of the development of a manic episode; (e) current treatment with more than four psychotropic medications; (f) medical illness including hepatic impairment, renal dysfunction, bleeding diatheses, cerebrovascular disease, hypertension or diabetes mellitus that is inadequately controlled by diet and/or medication, or known active peptic ulcer disease; (g) abuse of drugs or alcohol within the preceding 6 months, or substance dependence within the last year; (h) daily alcoholic beverage consumption equivalent to >3 oz. of alcohol; (i) known allergies or hypersensitivities to tetracycline antibiotics, aspirin or other NSAIDs; (j) current use of drugs that could increase the risks associated with aspirin or minocycline administration, (k) chronic infection, (l) use of antibiotics, (m) pregnant or nursing women, (n) asthma which in the opinion of the investigator would increase the likelihood of an asthmatic attack, and (o) regular use of steroidal or non-steroidal anti-inflammatory medications (occasional use of NSAIDS was allowed).

**Medical Assessment and Safety Screening**

The medical assessment included a physical examination, electrocardiogram, complete blood count (CBC), electrolytes and liver-function assays (SMA 20), thyroid panel, urinalysis, and urine pregnancy tests at the safety screening visit (i.e. prior to randomization), visit 4, and visit 7.

**Statistical Analysis**

There were no significant differences in baseline depression severity across the subject groups although the A+P group had a higher YMRS score at baseline (Table 1), the A+P and the P+P groups had higher concentrations of urine thromboxane B2 (table 1), and the M+A and P+P groups had a higher incidence of nervous system disorders (Table S1). Nevertheless, in the context of a randomized design it is not appropriate to attempt to statistically control for variables that differ by chance across groups ^1-4^ as illustrated in the current CONSORT guidelines (<http://www.consort-statement.org/consort-2010)>.

Linear mixed-effect (LME) model analyses were used in order to test the effects of treatment on the change in MADRS score. The analysis performed with nlme package ^5^ in R statistical language and environment ^6^. The LME model included the fixed effects of aspirin, minocycline, visit, and their interactions. Age, sex, and BMI were also included as fixed effects. Visit was entered as a factor variable following the mixed-effect model repeated measure (MMRM) approach ^7^, so that no linear change was assumed across visits. The MMRM approach controls for missing data due to dropout and avoids bias due to any imbalance in the number of visits between subjects.

Random effects were selected by a top-down approach ^8^, in which we started from a well-specified model and reduced it using the likelihood ratio test (LRT) and Akaike Information criterion (AIC). Initially, we modeled the random effects of subjects and study site on visit and intercept. This model, however, could not be identified due to over-parameterization. Subsequently we excluded a random effect of study site on visit time (model 1). This model was compared to a reduced model without study site as a random effect (model 2). Model 1 and model 2 did not differ in their likelihoods (log likelihood of model 1 = -1596.492, model 2 = -1596.492, LRT p = 1.0) and model 2 had a smaller AIC (model 1 = 3,292.983, model 2 = 3,290.983), indicating that model 2 was preferable to model 1. We further excluded the subject random effect on visit from model 2 (model 3). This model showed a significant reduction of likelihood (log likelihood of model 2 = -1596.492, model 3 = -1646.046735, LRT p < 0.001). Based on these results we therefore selected model 2, which included the subject random effect on visit time and intercept.

We also considered the autocorrelation noise in residual variance-covariance matrix structure since the time-course of the change in the MADRS score can be autocorrelated within a subject and thus modeling autocorrelation is recommended in the MMRM approach ^7^. We therefore added the AR(1) residual correlation model into model 2 (model 4). This model, however, did not improve likelihood (log likelihood of model 2 = -1596.492, model 4 = -1596.492, LRT p = 1.0), and additionally possessed larger AIC than models 2 and 3 (model 2 = 3,290.983, model 3 = 3,292.983). Based on this result, we did not include autocorrelation residual structure into the model. The same approach was taken for other LME models that included the fixed effect of cytokines. For all LME evaluations, the random effect of model 2 (subject random effect on visit time and intercept without autocorrelation residual structure) was preferred.

The significance of each fixed effect in the LME model was examined with an analysis of deviance likelihood ratio test that evaluates the likelihood ratio between models with and without a testing term ^9^. A type-II approach was used, in which each term was tested after all other effects (except the term's higher-order relatives) were controlled ^9^.

**Supplementary Tables**

Table S1. Psychiatric History, Medical Comorbidity, and Medication Status of the Randomized Participants.

|  | M+A | M+P | P+A | P+P | $\boldsymbol{\chi}\boldsymbol{2}$ | P |
| --- | --- | --- | --- | --- | --- | --- |
| N | 31 | 19 | 19 | 30 |  |  |
| Psychiatric History^1^ |  |  |  |  |  |  |
| Family Psychiatric History (Any) - frequency (%) | 21 (91.3) | 16 (88.9) | 18 (100.0) | 27 (90.0) | 1.97 | 0.579 |
| No information | 8 | 1 | 1 | - |  |  |
| Comorbid Physical History (Current) |  |  |  |  |  |  |
| Current smoker - frequency (%) | 9 (29.0) | 7 (43.8) | 3 (18.8) | 13 (50.0) | 4.75 | 0.191 |
| Nervous system disorder - frequency (%) ß | 23 (74.2) | 12 (63.2) | 8 (42.1) | 23 (76.7) | 8.03 | 0.045* |
| Respiratory system disorder - frequency (%) Ω | 15 (48.4) | 5 (26.3) | 9 (47.4) | 11 (36.7) | 3.25 | 0.354 |
| Cardiovascular disorder - frequency (%) ∆ | 8 (25.8) | 5 (26.3) | 4 (21.1) | 12 (40.0) | 2.45 | 0.485 |
| Endocrine - frequency (%) µ | 12 (38.7) | 7 (38.9) | 8 (42.1) | 9 (30.0) | 0.98 | 0.805 |
| Gastrointestinal disorder - frequency (%) ∂ | 6 (19.4) | 6 (31.6) | 6 (31.6) | 14 (46.7) | 2.91 | 0.405 |
| Genitourinary disorder - frequency (%) ∑ | 3 (9.7) | 1 (5.3) | 2 (10.5) | 5 (16.7) | 1.52 | 0.678 |
| Musculoskeletal disorder - frequency (%) ƒ | 18 (58.1) | 14 (73.7) | 14 (73.7) | 23 (76.7) | 0.34 | 0.952 |
| Comorbid - other - frequency (%) π | 23 (74.2) | 16 (84.2) | 15 (78.9) | 27 (90.0) | 2.09 | 0.553 |
| Baseline Medication |  |  |  |  |  |  |
| Antidepressant - frequency (%) | 11 (36.7) | 9 (47.4) | 10 (52.6) | 17 (56.7) | 2.61 | 0.455 |
| Anxiolytics - frequency (%) | 6 (19.4) | 5 (26.3) | 7 (36.8) | 6 (20.0) | 2.01 | 0.570 |
| Antipsychotic - frequency (%) | 9 (30.0) | 10 (52.6) | 8 (42.1) | 10 (33.3) | 2.96 | 0.398 |
| Mood stabilizer - frequency (%) | 8 (26.7) | 6 (31.6) | 6 (31.6) | 10 (33.3) | 0.34 | 0.952 |
| Complimentary (including multi-vitamin - frequency (%) | 4 (13.3) | 5 (26.3) | 4 (21.1) | 5 (16.7) | 1.46 | 0.692 |
| Other - frequency (%) | 21 (70.0) | 14 (73.7) | 13 (68.4) | 21 (70) | 0.14 | 0.987 |
| Comorbid Diagnosis (DSM-IV) |  |  |  |  |  |  |
| Dysthymic disorder - frequency (%)^2^ | 1 (3.2) | 1 (5.3) | 0 (0.0) | 0 (0.0) | 2.25 | 0.523 |
| Anxiety disorders (pooled) - current^ - frequency (%)^3^ | 5 (16.1) | 5 (26.3) | 4 (21.1) | 10 (33.3) | 2.61 | 0.456 |
| Post-traumatic stress disorder - frequency (%)^4^ | 7 (22.6) | 4 (21.1) | 3 (15.8) | 3 (10.0) | 1.95 | 0.583 |
| Eating disorder - frequency (%)^5^ | 2 (6.5) | 1 (5.3) | 1 (5.3) | 1 (3.3) | 0.31 | 0.957 |
| Obsessive compulsive disorder - frequency (%)^6^ | 1 (3.2) | 1 (5.3) | 1 (5.3) | 1 (3.3) | 0.24 | 0.971 |

* p<0.05, uncorrected.

^1^Total frequency, includes multiple medication types for the same participant

^2^Includes 300.04: Dysthymic Disorder

^3^Includes 300.0, 300.02, 300.21: Anxiety Disorder NOS; Generalized Anxiety Disorder; Panic Disorder with Agoraphobia

^4^Includes 309.81: Post-traumatic Stress Disorder

^5^Includes 307.1, 307.5, 307.51: Anorexia Nervosa; Eating Disorder NOS; Bulimia Nervosa

^6^Includes 300.3: Obsessive Compulsive Disorder

ß – one or more of the following conditions: head injury; concussion; loss of consciousness; loss of memory or amnesia; migraine headaches or frequent or recurring headaches; brain tumor; light-headedness or dizziness; frequent fainting spells; epilepsy or seizures; stroke; tingling sensation; neuritis or numbness; paralysis; other (e.g. multiple sclerosis).

Ω - one or more of the following conditions: chronic or frequent cough; chronic or frequent colds; tuberculosis; asthma; wheezing; bronchitis; shortness of breath; sleep apnea; pneumonia; spitting up blood; emphysema or chronic obstructive pulmonary disease (COPD).

∆ – one or more of the following conditions: heart disease; heart murmur; mitral valve prolapse; scarlet fever; rheumatic fever; heart surgery; congestive heart failure; pain or pressure in chest; palpitations; irregular pulse; history of heart attack.

µ – one or more of the following conditions: thyroid disease; kidney disease; diabetes; hypoglycemia; hormonal disorders.

∂ – one or more of the following conditions: abdominal pain; nausea and vomiting; intestinal problems; frequent diarrhea; hepatitis; celiac disease; blood in stool; stomach ulcer; vomiting blood; history of liver disease; jaundice; esophageal varices; ulcerative colitis.

∑ – one or more of the following conditions: frequent or painful urination; blood in urine; sugar or albumin in urine; kidney stones.

ƒ – one or more of the following conditions: bone, joint or other deformity; juvenile arthritis; shoulder or elbow pain; knee pain; muscle or joint weakness; recurrent back pain; arthritis, rheumatism or bursitis; broken bones; swollen or painful joints.

π – one or more of the following conditions: fever; recent weight changes; blurred or double vision; eye disease; glaucoma; cataracts; severe tooth or gum problems; hearing loss or ringing; chronic sinus problems; nose bleeds; sore throat or voice change; allergies; skin disease; rash; enlarged glands; anemia; HIV; cancer; syphilis.

Table S2. Summary of Potential Side-Effects Recorded.

| Adverse Event | P+P | P+A | M+P | M+A |
| --- | --- | --- | --- | --- |
| Nausea | 1 | 0 | 0 | 2 |
| Vomiting | 2 | 0 | 0 | 0 |
| Suicidal Ideation | 0 | 0 | 1 | 0 |
| Transient Ischemic Attack | 0 | 1 | 0 | 0 |
| Heart palpitations | 0 | 1 | 0 | 0 |
| Dizziness | 0 | 1 | 0 | 0 |
| Reduced K+ | 1 | 0 | 0 | 0 |
| Vaginal itching | 0 | 0 | 0 | 1 |
| Vaginal discharge | 0 | 0 | 0 | 1 |
| "Food poisoning" | 0 | 0 | 0 | 1 |
| Heart burn | 0 | 0 | 0 | 1 |
| Burning/itching during urination | 0 | 0 | 0 | 1 |
| Headache | 0 | 0 | 1 | 0 |
| Bacterial vaginosis | 0 | 0 | 1 | 0 |
| Homicidal Ideation | 0 | 1 | 0 | 0 |
| Hot/cold sensations | 1 | 0 | 0 | 0 |
| Myalgia | 1 | 0 | 0 | 0 |
| Diarrhea | 0 | 0 | 0 | 1 |
| Rash | 1 | 0 | 0 | 0 |
| Hypomania | 0 | 0 | 0 | 1 |

**Table S3. Summary of the Principal Results from Primary and Secondary Hypotheses**

| Primary Hypothesis Tested | Result | Main Interpretation | Related Figure |
| --- | --- | --- | --- |
| 1. *Response*: M+A versus P+P | X_1_^2^=3.35, p(1t)=0.034, OR=2.93, NNT=4.7 | Patients receiving M+A were more likely to respond to treatment than patients receiving P+P | 2 |
| Secondary Hypotheses Tested |  |  |  |
| 2. *Response*: Effect of M and A across all four study arms | Significant effect of A (X_1_^2^=5.52, p(2t)=0.019, OR=3.67)  NNT (M+A and A+P vs M+P and P+P) = 4.2  NNT (M+A and A+P vs P+P) = 4.0  No significant effect of M  No interaction between M and A | Patients receiving A significantly more likely to respond to treatment than patients not receiving A | 2 |
| 3a. *Response:* Levels of baseline inflammation will be associated with response to A and M | Three-way interaction between M, A, and IL-6 (X^2^_1_=7.08, p(2t)=0.008)  Follow-up analysis:  M+P with higher IL-6 responded better than M+P with lower IL-6 (X^2^_1_=7.72, p(2t)=0.005) | M may be more efficacious in people with greater inflammation | 3 |
| 3b. *Response:* Participants who show a greater decrease in IL-6 and CRP concentrations between V1 and V7 will show a greater response rate | Significant interaction between the change in IL-6 and treatment response (X^2^_3_=9.69, p(2t)=0.001) | Participants in the M+P group who responded to treatment showed a significant decrease in IL-6 concentrations between V1 and V7 | - |

------------------------------------------------------------------------------------------------------------------------------------------------------------------

A = aspirin; M = minocycline; A+P = aspirin plus placebo; M+P = minocycline plus placebo; M+A = minocycline plus aspirin; NS=not significant; OR = odds ratio; NNT = number needed to treat; IL-6 = interleukin 6; CRP = C-reactive protein; MADRS = Montgomery Äsberg Depression Rating Scale; V1 = visit 1 (baseline); V7 = visit 7 (final visit); q=corrected p-value; p(1t) = p-value from 1 tailed test; p(2t) = p-value from 2-tailed test.

------------------------------------------------------------------------------------------------------------------------------------------------------------------

**References**

1. de Boer MR, Waterlander WE, Kuijper LD, Steenhuis IH, Twisk JW. Testing for baseline differences in randomized controlled trials: an unhealthy research behavior that is hard to eradicate. *Int J Behav Nutr Phys Act* 2015; **12:** 4.

2. Senn S. Testing for baseline balance in clinical trials. *Stat Med* 1994; **13**(17)**:** 1715-1726.

3. Altman DG. Comparability of randomised groups. *Statistician* 1985; **34:** 125-136.

4. Altman DG, Dore CJ. Randomisation and baseline comparisons in clinical trials. *Lancet* 1990; **335**(8682)**:** 149-153.

5. Pinheiro J, Bates D, DebRoy S, Sarkar D, R Core Team. nlme: Linear and Nonlinear Mixed Effects Models. 2017; **R package version 3.1-131**.

6. R: A Language and Environment for Statistical Computing. [http://www.r-project.org/](http://www.R-project.org), 2017, Accessed Date Accessed 2017 Accessed.

7. Siddiqui O, Hung HM, O'Neill R. MMRM vs. LOCF: a comprehensive comparison based on simulation study and 25 NDA datasets. *Journal of biopharmaceutical statistics* 2009; **19**(2)**:** 227-246.

8. West BT, Galecki AT, Welch KB. *Linear mixed models: A Practical Guide Using Statistical Software, Second Edition*. Chapman and Hall/CRC2014.

9. Fox J, Weisberg HS. *An R Companion to Applied Regression, Second Edition*. Sage2011, 472pp.
