# Supplementary figures and images for: Treatment of bipolar depression with minocycline and/or aspirin: an adaptive, 2×2 double-blind, randomized, placebo-controlled, phase IIA clinical trial
Source: Transl Psychiatry. 2018 Jan 24;8:27. doi: 10.1038/s41398-017-0073-7 (PMC5802452; doi:10.1038/s41398-017-0073-7)

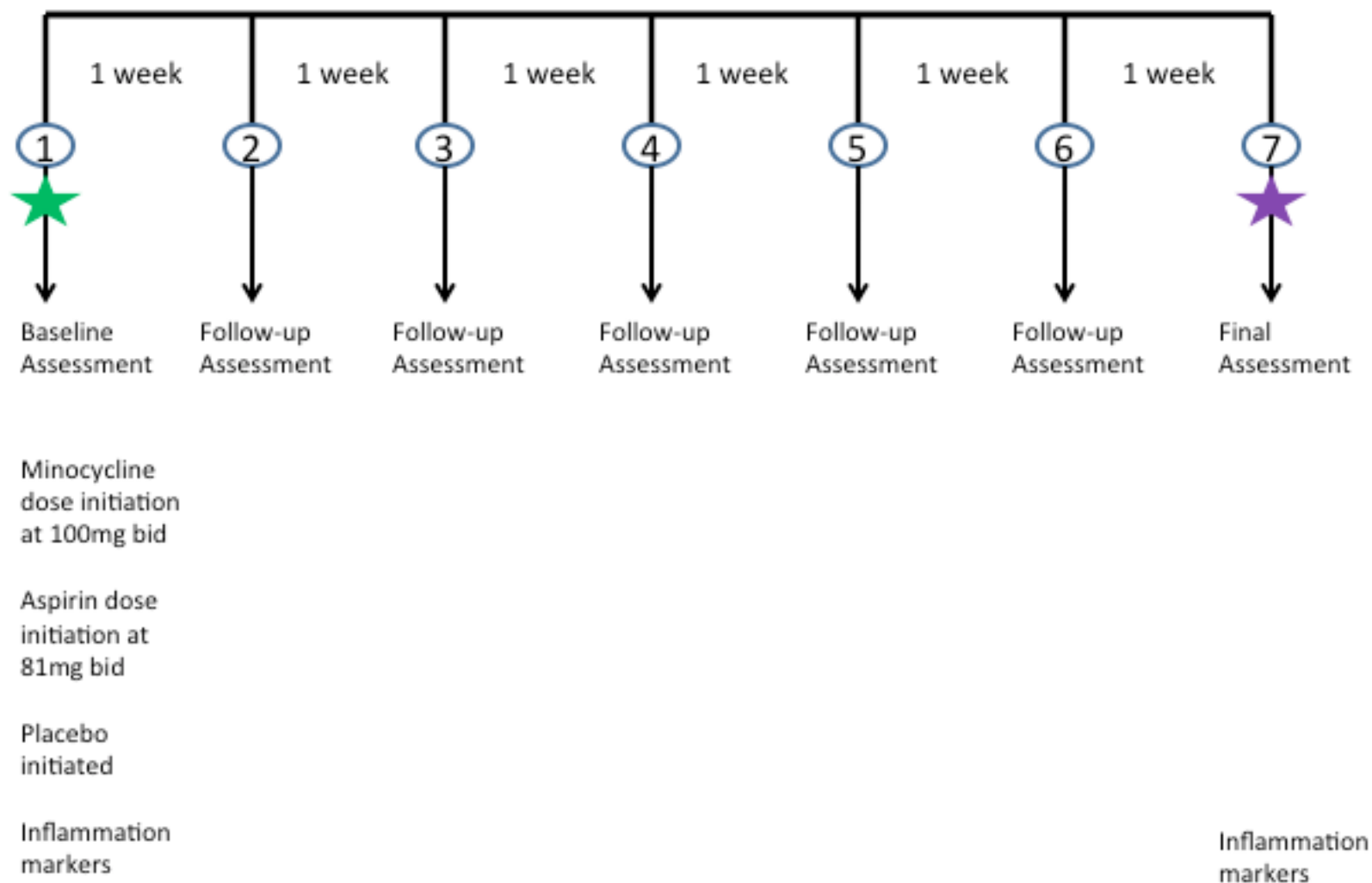

Figure S1

Supplement: Supplementary file 2 — Supplementary FigureS1 [file 41398_2017_73_MOESM2_ESM.pdf]

Change in thromboxane B2 for each individual after treatment

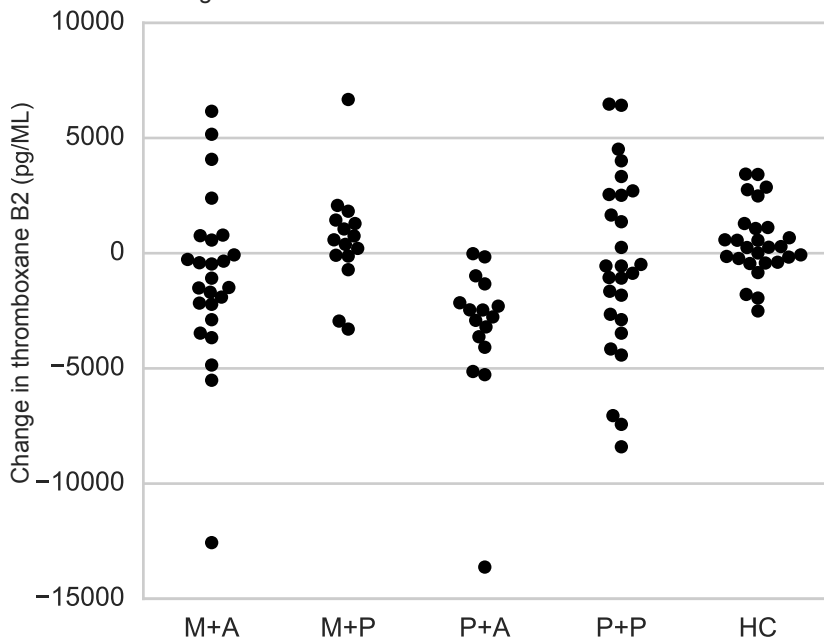

Figure S2

Supplement: Supplementary file 3 — Supplementary FigureS2 [file 41398_2017_73_MOESM3_ESM.pdf]

A

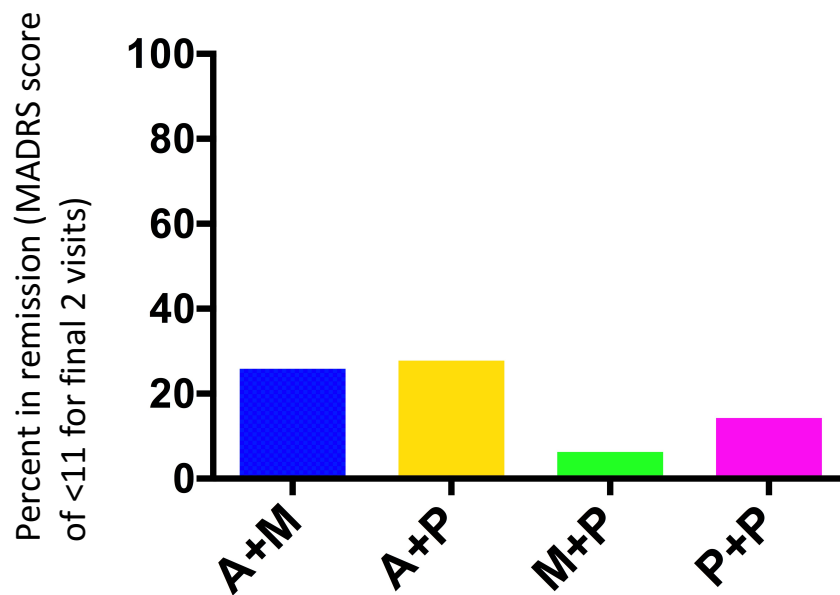

B

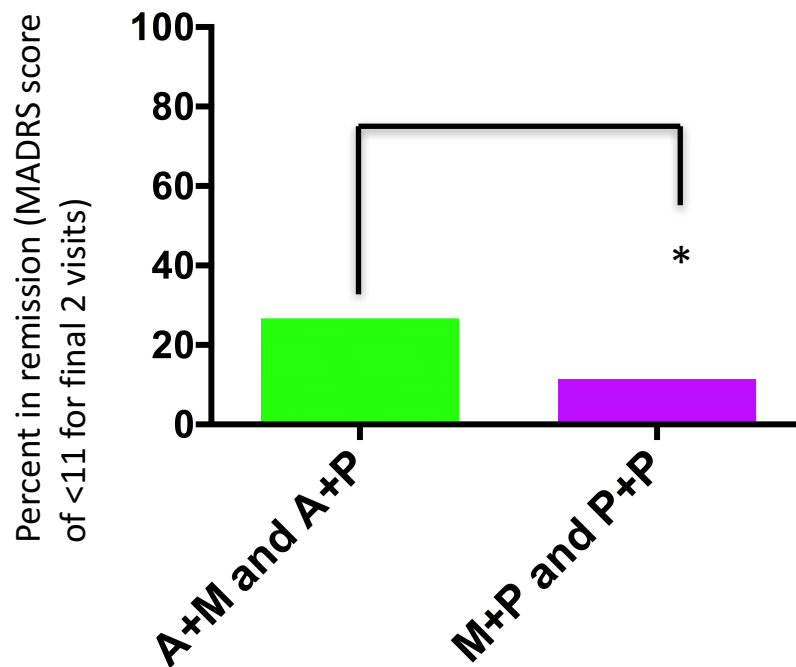

Figure S3

Supplement: Supplementary file 4 — Supplementary FigureS3 [file 41398_2017_73_MOESM4_ESM.pdf]

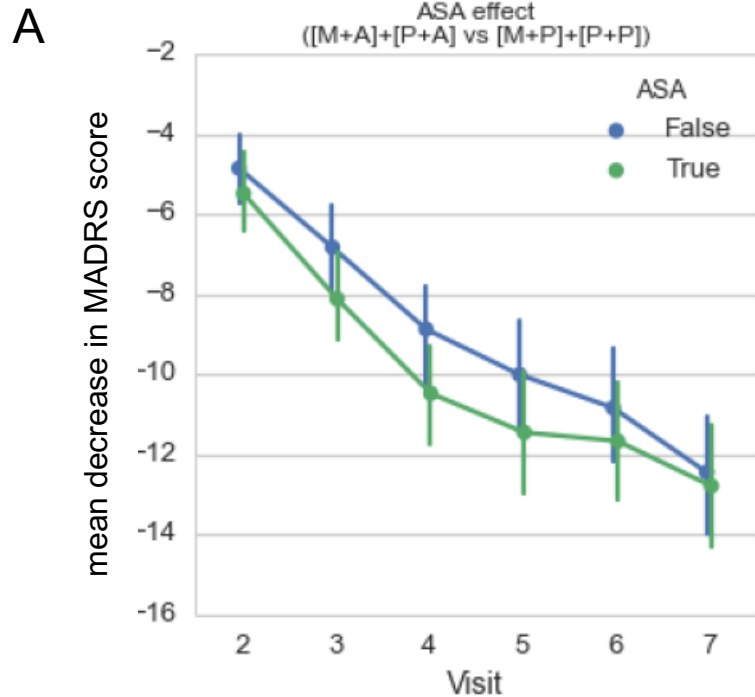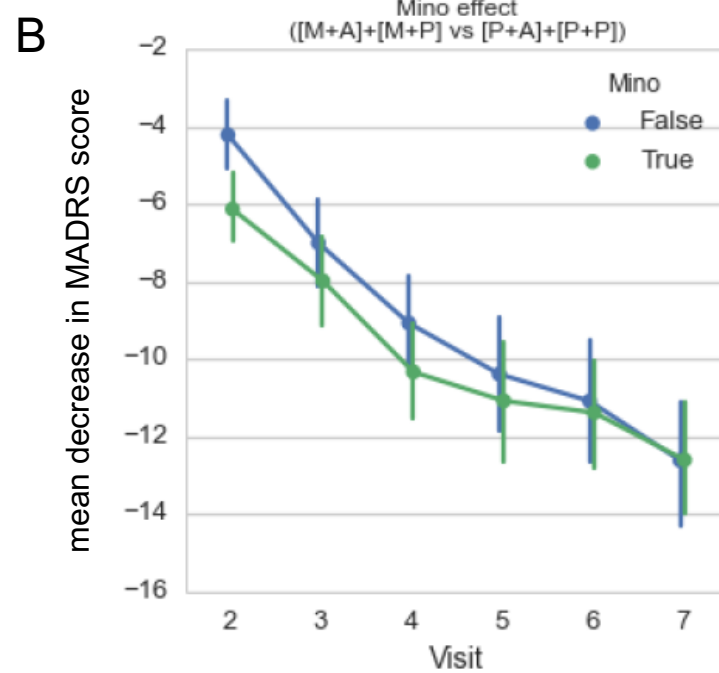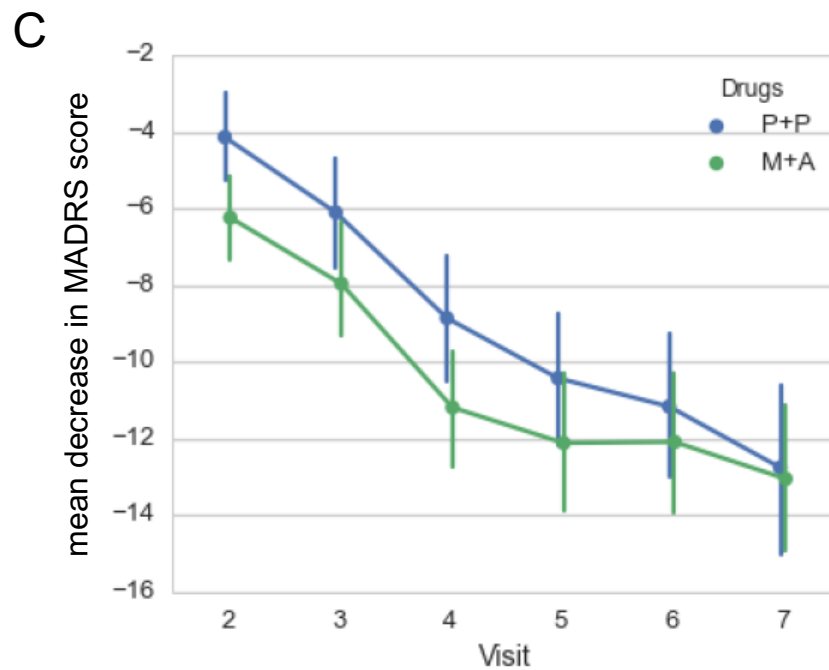

Figure S4

Supplement: Supplementary file 5 — Supplementary FigureS4 [file 41398_2017_73_MOESM5_ESM.pdf]

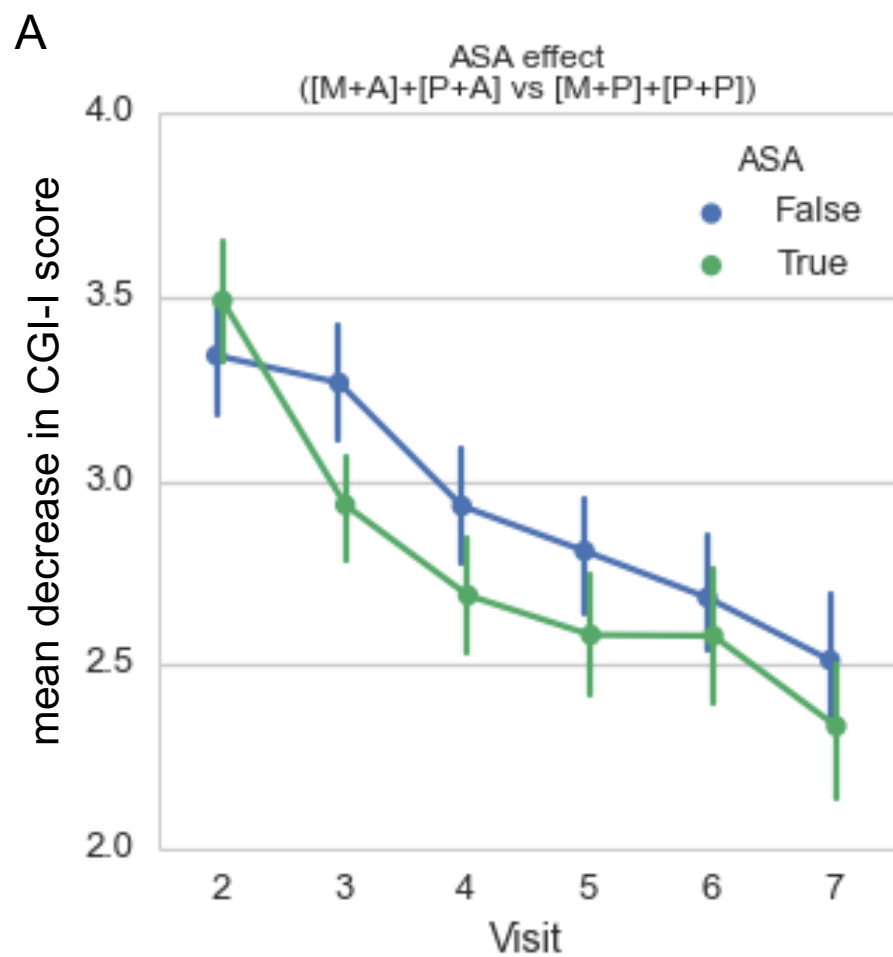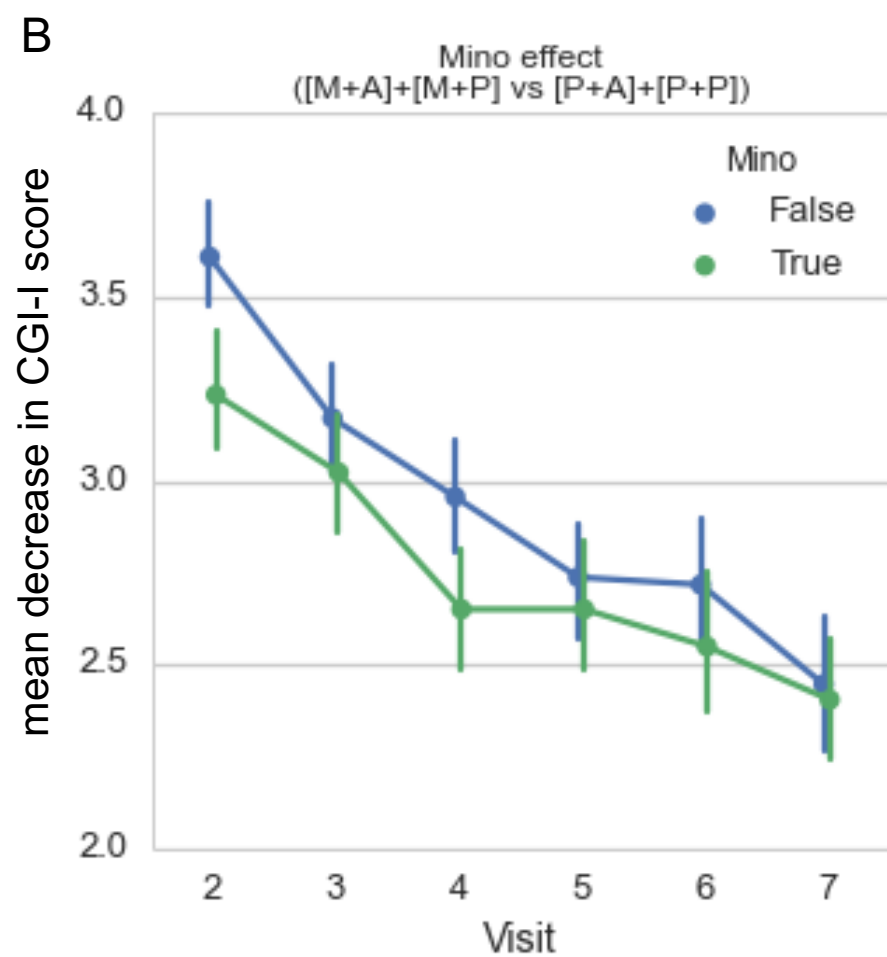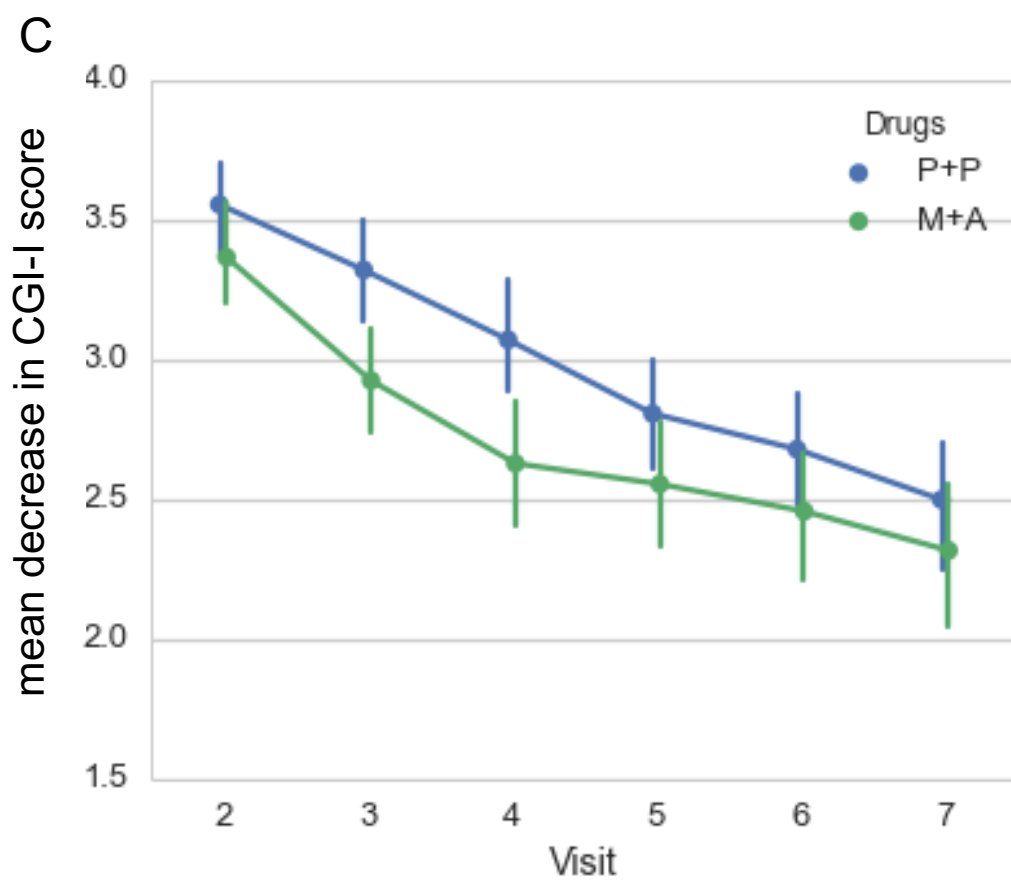

Figure S5

Supplement: Supplementary file 6 — Supplementary FigureS5 [file 41398_2017_73_MOESM6_ESM.pdf]

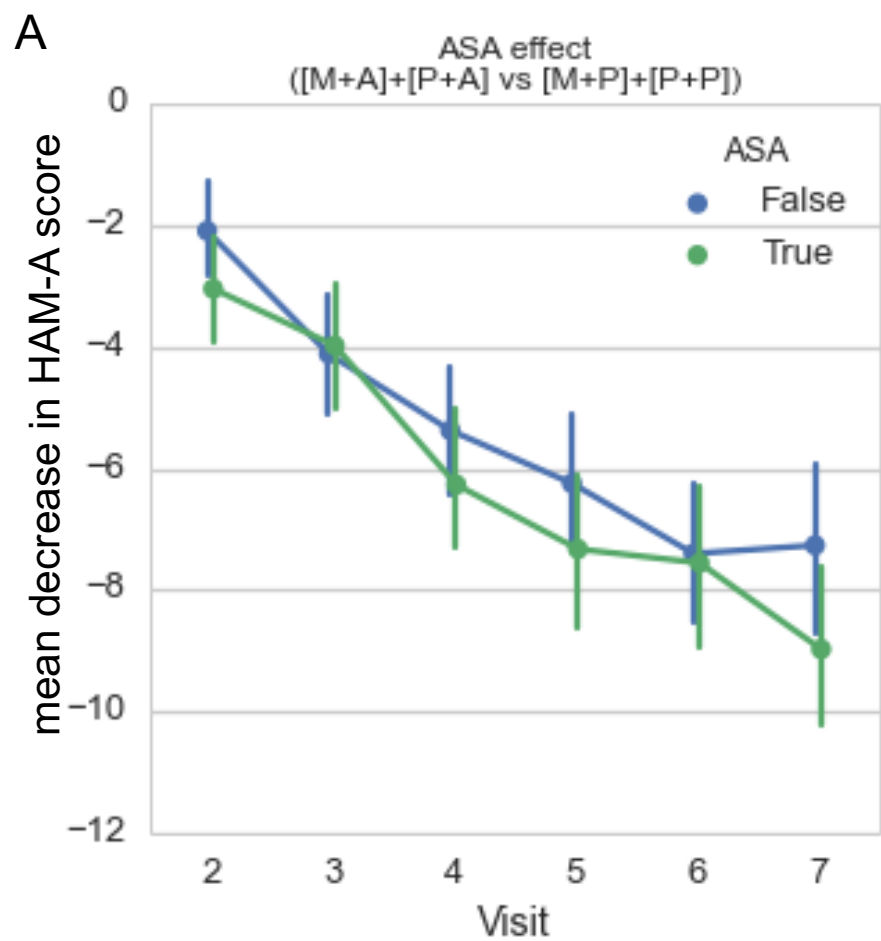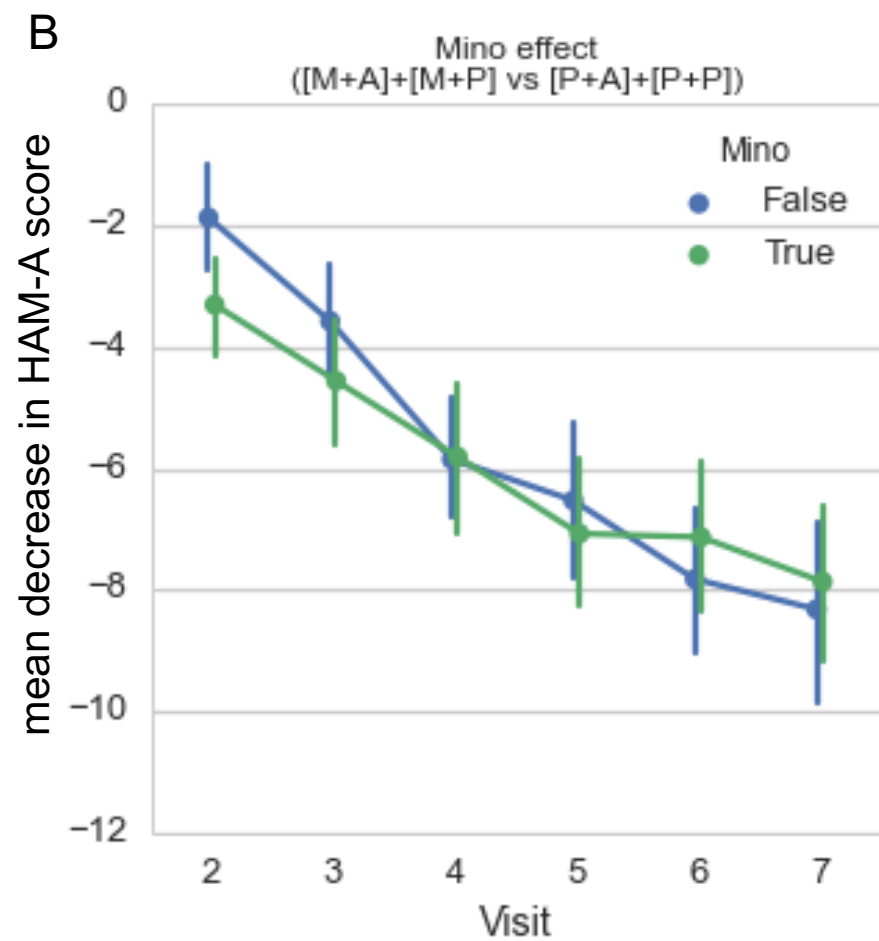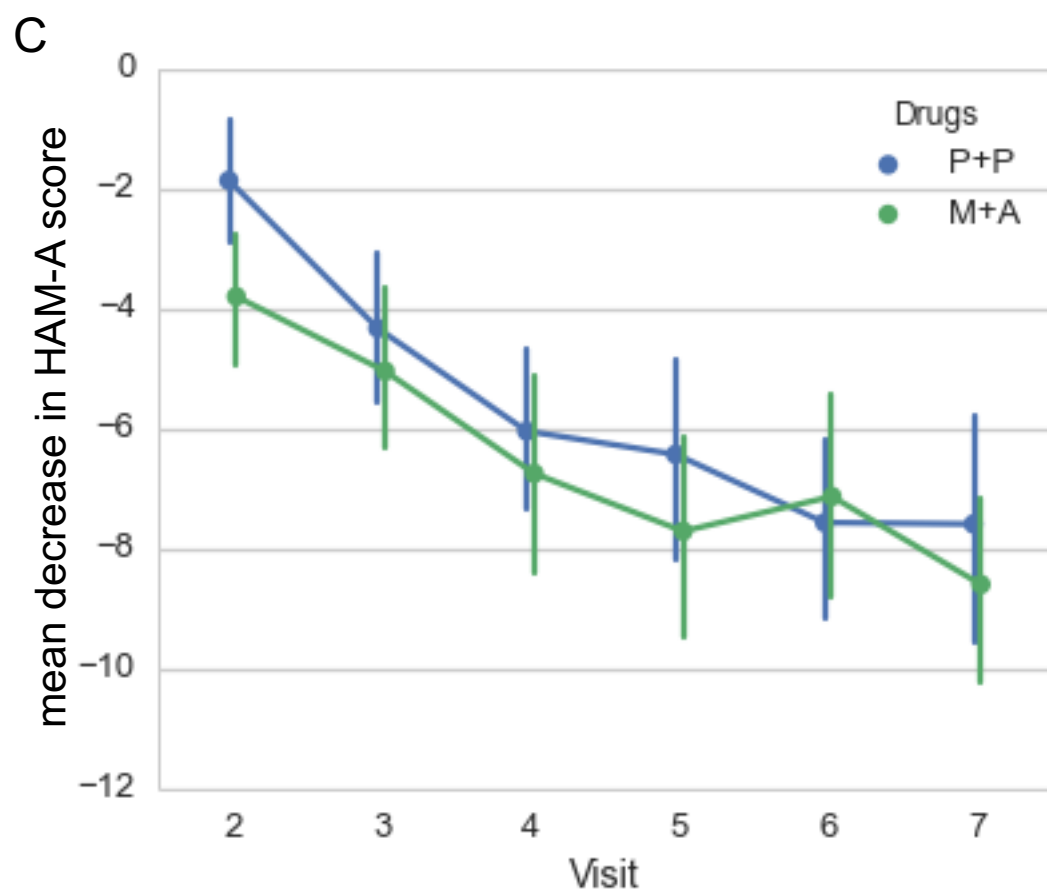

Figure S6

Supplement: Supplementary file 7 — Supplementary FigureS6 [file 41398_2017_73_MOESM7_ESM.pdf]
