# Supplementary material for: Treatment of bipolar depression with minocycline and/or aspirin: an adaptive, 2×2 double-blind, randomized, placebo-controlled, phase IIA clinical trial
Source: Transl Psychiatry. 2018 Jan 24;8:27. doi: 10.1038/s41398-017-0073-7 (PMC5802452; doi:10.1038/s41398-017-0073-7)
Supplement: Supplementary file 8 — Supplementary Figure Legends [file 41398_2017_73_MOESM8_ESM.docx]

**Supplementary Figure Legends**

Figure S1: Each session number (total of 7) is encircled, with the timing between sessions indicated in weeks with a two business day window on either side of visit target date to complete the visit. Session 1 is the baseline (green star) and session 7 is the study end (purple star). The study duration is 6 weeks.

Figure S2: Plot showing the change (visit 1 versus visit 7) in urine 11-D-TXB_2_ concentrations for each individual across the four treatment groups and healthy controls. Points above the x-axis indicate increases in 11-D-TXB_2_ concentration over time, while points below the x-axis indicate decreases in 11-D-TXB_2_ concentrations over time. The mean change in 11-D-TXB_2_ for each group was as follows: -1068±3696 (M+A), 606±2277 (M+P), -3281±3147 (A+P), -473±3918 (P+P), and 468±1516 (HC). There was a significant decrease between visit 1 and visit 7 in 11-D-TXB_2_ concentrations in the A+P group (t_15_=4.2, p=0.001) but not the M+A group (t_24_=1.4, p=0.162). Interestingly, baseline concentrations of (log-normalized) 11-D-TXB_2_ were significantly higher in the entire BD group compared with the healthy control group (t_43_=2.43, p(2t)=0.019). The baseline concentrations of 11-D-TXB_2_ were as follows: M+A (2,665±2,834 pg/mL); M+P (2,730±2,449 pg/mL); A+P (4,765±3,704 pg/mL); P+P (5,038±4,626); combined BD sample (3,809±3,679), and HC (2,051±1,880 pg/mL).

Note that the healthy control did not receive treatment with minocycline and/or aspirin.

Figure S3: Percentage of remitters (y-axis) in each of the four treatment groups shown individually (A, top panel) and the two aspirin groups (M+A and A+P) versus the two non-aspirin groups (M+P and P+P) (B, bottom panel). Top panel: there was no statistically significant difference in remission rate between participants in the four treatment arms. Bottom panel: there was a main effect for aspirin on the remission rate that did not remain significant after Bonferroni correction (X_1_^2^=4.14, q(2t)>0.5, p(2t)=0.042, uncorrected, OR=2.52, CI=0.56-12.29). There was no main effect for minocycline (X_1_^2^=0.45, p=0.503, uncorrected) or interaction between aspirin and minocycline (X_1_^2^=0.35, p=0.554, uncorrected). The NNT for aspirin to obtain remission (M+A and A+P versus M+P and P+P) was 6.5. The NNT for the M+A and A+P versus P+P comparison was 8.0.

* p<0.05 (two-tailed test)

Figure S4: The mean decrease in the Montgomery Depression Rating Scale (MADRS) score (y-axis) across visits (x-axis) is shown for: (A) the combined aspirin groups versus the non-aspirin groups (i.e. the main effect of aspirin), (B) the combined minocycline groups versus the non-minocycline groups (i.e. the main effect of minocycline), and (C) the M+A group versus the P+P group. There were no significant effects of aspirin (F_1,87_=1.04, p=0.311, shown in panel A), minocycline (F_1,87_=0.22, p=0.638, shown in panel B) or M+A (F_1,52_=1.14, p=0.291, shown in panel C).

The error bars represent the standard error of the mean. All p-values are uncorrected.

Figure S5: The mean decrease in the Clinical Global Impression Improvement (CGI-I) score (y-axis) across visits (x-axis) is shown for: (A) the combined aspirin groups versus the non-aspirin groups (i.e. the main effect of aspirin), (B) the combined minocycline groups versus the non-minocycline groups (i.e. the main effect of minocycline), and (C) the M+A group versus the P+P group. There were no significant effects of aspirin (F_1,87_=1.09, p=0.300, shown in panel A), minocycline (F_1,87_=0.63, p=0.428, shown in panel B) or M+A (F_1,52_=1.58, p=0.214, shown in panel C).

The error bars represent the standard error of the mean. All p-values are uncorrected.

Figure S6: The mean decrease in the Hamilton Anxiety Scale (HAM-A) score (y-axis) across visits (x-axis) is shown for: (A) the combined aspirin groups versus the non-aspirin groups (i.e. the main effect of aspirin), (B) the combined minocycline groups versus the non-minocycline groups (i.e. the main effect of minocycline), and (C) the M+A group versus the P+P group. There were no significant effects of aspirin (F_1,87_=0.28, p=0.597, shown in panel A), minocycline (F_1,87_=0.12, p=0.734, shown in panel B) or M+A (F_1,52_=1.14, p=0.291, shown in panel C).

The error bars represent the standard error of the mean. All p-values are uncorrected.
